# Synergistic Antibiofilm Action of *Cinnamomum verum* and Brazilian Green Propolis Hydroethanolic Extracts against Multidrug-Resistant Strains of *Acinetobacter baumannii* and *Pseudomonas aeruginosa* and Their Biocompatibility on Human Keratinocytes

**DOI:** 10.3390/molecules28196904

**Published:** 2023-10-01

**Authors:** Vanessa Marques Meccatti, Karoline Moura Chagas Martins, Lucas de Paula Ramos, Thaís Cristine Pereira, Raquel Teles de Menezes, Maria Cristina Marcucci, Amjad Abu Hasna, Luciane Dias de Oliveira

**Affiliations:** 1 Department of Biosciences and Oral Diagnosis, Institute of Science and Technology, São Paulo State University (ICT-UNESP), São José dos Campos 12245-000, SP, Brazil; vanessameccatti@gmail.com (V.M.M.); cris.marcucci@yahoo.com.br (M.C.M.); luciane.oliveira@unesp.br (L.D.d.O.); 2 Department of Restorative Dentistry, Endodontics Division, Institute of Science and Technology, São Paulo State University (ICT-UNESP), São José dos Campos 12245-000, SP, Brazil

**Keywords:** *Acinetobacter baumannii*, *Cinnamomum verum*, HaCaT Cells, propolis, *Pseudomonas aeruginosa*

## Abstract

The accumulated dental biofilm can be a source of oral bacteria that are aspirated into the lower respiratory tract causing ventilator-associated pneumonia in hospitalized patients. The aim of this study was to evaluate the synergistic antibiofilm action of the produced and phytochemically characterized extracts of *Cinnamomum verum* and Brazilian green propolis (BGP) hydroethanolic extracts against multidrug-resistant clinical strains of *Acinetobacter baumannii* and *Pseudomonas aeruginosa*, in addition to their biocompatibility on human keratinocyte cell lines (HaCaT). For this, High-performance liquid chromatography analysis of the plant extracts was performed; then the minimum inhibitory and minimum bactericidal concentrations of the extracts were determined; and antibiofilm activity was evaluated with MTT assay to prevent biofilm formation and to reduce the mature biofilms. The cytotoxicity of the extracts was verified using the MTT colorimetric test, evaluating the cellular enzymatic activity. The data were analyzed with one-way ANOVA and Tukey’s tests as well as Kruskal–Wallis and Dunn’s tests, considering a significance level of 5%. It was possible to identify the cinnamic aldehyde in *C. verum* and p-coumaric, caffeic, and caffeoylquinic acids as well as flavonoids such as kaempferol and kaempferide and Artepillin-C in BGP. The combined extracts were effective in preventing biofilm formation and reducing the mature biofilms of *A. baumannii* and *P. aeruginosa*. Moreover, both extracts were biocompatible in different concentrations. Therefore, C. *verum* and BGP hydroethanolic extracts have bactericidal and antibiofilm action against multidrug resistant strains of *A. baumannii* and *P. aeruginosa*. In addition, the combined extracts were capable of expressively inhibiting the formation of *A. baumannii* and *P. aeruginosa* biofilms (prophylactic effect) acting similarly to 0.12% chlorhexidine gluconate.

## 1. Introduction

Multidrug-resistant bacteria pose a critical threat to global health, worsening prognosis and increasing mortality among hospitalized infected patients [1]. In 2017, the World Health Organization (WHO) published the document “priority pathogens” which cites the main multidrug-resistant bacteria responsible for nosocomial infections, guiding the research and development of new antimicrobials and antibiotics in which *Pseudomonas aeruginosa* and *Acinetobacter baumannii* were considered critical microorganisms (Priority 1) according to the document [2]. *P. aeruginosa* and *A. baumannii* are Gram-negative, largely biofilm-forming bacteria and have a high incidence of resistance to antibiotics, especially to carbapenems [3]. *A. baumannii* quickly evolved as a nosocomial pathogen and is currently considered as a serious threat to global health [4]. 

Oral bacteria are accumulated in dental calculus, which is a calcified oral plaque biofilm, and can be aspirated into the lower respiratory tract causing ventilator-associated pneumonia in hospitalized patients [5]; this pneumonia may be caused by *A. baumannii* with mortality rates ranging from 40 to 70% [6] or by *P. aeruginosa* [7]. Still, an increased risk of co-infection by these and other opportunistic microorganisms was reported in Coronavirus disease (COVID-19) patients as the severe acute respiratory syndrome coronavirus 2 (SARS-CoV-2) has strategies to disrupt immune mechanisms [8].

Chlorhexidine digluconate (CHX) is an antiseptic mouthwash. It is indicated for the oral hygiene control of hospitalized patients in intensive care units; however, its efficacy is controversial [9,10]. Moreover, it causes tooth staining, taste alteration and oral mucosa irritation [11]. Therefore, the development of new effective biocompatible antimicrobial substances with fewer side effects is strongly recommended to be used as antiseptic mouthwashes. In this context, some herbal plant extracts were found effective against multidrug-resistant bacteria and mixed biofilms [12,13,14], as plants generate secondary metabolites for their own defense against environmental challenges, which results in several molecules with antibacterial mechanisms comparable to traditional antibiotics [15].

*Cinnamomum verum*, popularly known as Cinnamon, is a small evergreen tree belonging to the family Lauraceae, native to Sri Lanka [16]. The inner bark of several other trees of the genus *Cinnamomum* are used to obtain cinnamon which has a wide variety of secondary metabolites that exhibit antibacterial properties, such as cinnamaldehyde or cinnamic acid. Products derived from cinnamon can inhibit the cell division and biofilm formation of several bacteria with mechanisms such as a negative regulation of genes associated with motility and quorum detection systems, as well as an inhibition of polysaccharide synthesis including that of *Pseudomonas* spp. [17]. Furthermore, propolis, a resinous substance enriched by biologically active molecules and produced by *Apis mellifera* through the collection of different plant parts, has different types according to the color and geographic region, in which the Brazilian green propolis botanic source is *Baccharis dracunculifolia’s* DC, popularly known as ‘alecrim-do-campo’, a plant from the *Asteraceae* family [18]. Among the vast chemical composition of propolis, derivatives of cinnamic acid are mentioned, such as p-coumaric acid and derivatives, other phenolic acids, flavonoids and amino acids [19]. Its antimicrobial and antibiofilm properties are extensively reported against several microorganisms, including multidrug-resistant strains of *P. aeruginosa* and *Klebsiella pneumoniae*, and against dental anaerobic bacteria, including *Fusobacterium nucleatum*, *Parvimonas micra*, *Prevotella intermedia*, *Porphyromonas gingivalis* and *Porphyromonas endodontalis* [12,20].

The synergy effect of two or more different materials can occur through several mechanisms: pharmacodynamic synergism, pharmacokinetic synergism, elimination of adverse effects and bypassing resistance mechanisms [21]. Thus, microbial resistance is less likely to occur when using combined compounds than against individual active components. Therefore, the aim of the study was to evaluate the synergistic antibiofilm action of the produced and phytochemically characterized extracts of *C. verum* and BGP propolis against multidrug-resistant clinical strains of *A. baumannii* and *P. aeruginosa*, in addition to their biocompatibility on human keratinocyte cell lines (HaCaT). The null hypothesis is that the extracts have no antimicrobial effect against *A. baumannii* and *P. aeruginosa* and are cytotoxic.

## 2. Results

### 2.1. Content of Soluble Solids

The content of soluble solids of *C. verum* hydroethanolic extract was 35.9 mg/mL, and that of Brazilian green propolis (BGP) hydroethanolic extract was 80.4 mg/mL.

### 2.2. High-Performance Liquid Chromatography (HPLC) Analysis 

**Figure 1:**
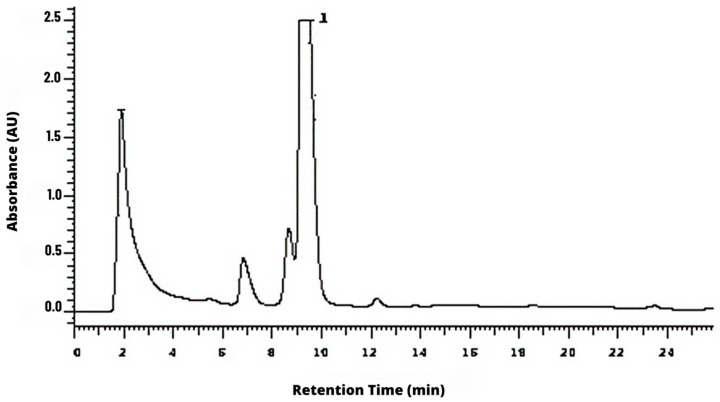
Chemical characterization of *C. verum* hydroethanolic extract according to high-performance liquid chromatography (HPLC) methods at wavelength of 280 nm. Peak # **1** is cinnamic aldehyde. AU corresponds to Absorbance Units.

**Figure 2:**
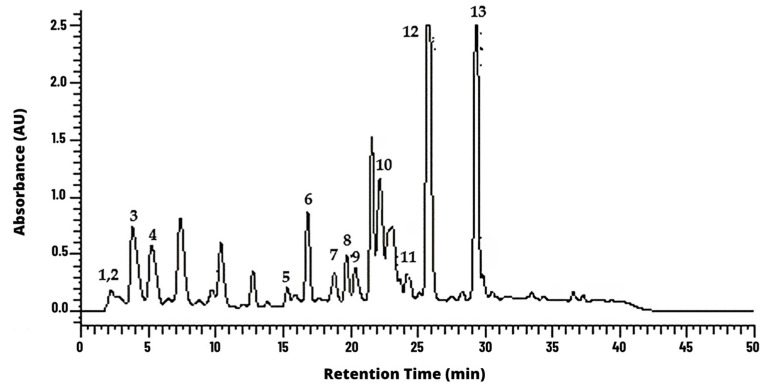
Chemical characterization of BGP hydroethanolic extract according to high-performance liquid chromatography (HPLC) methods at wavelength of 280 nm. Peaks are as follows: monocaffeoylquinic acid (**1**), caffeic acid (**2**), p-coumaric acid (**3**), 3,5-di-*O*-caffeoylquinic acid (**4**), kaempferol (**5**), 2,2-dimethyl-2H-1-benzopyranpropenoic acid (**6**), 3-prenyl-4-hydroxycinnamic acid (**7**), 4-hydroxy-3(E)-(4-hydroxy-3-methyl-2-buthenyl)-5-prenylcinnamic acid (**8**), 3-prenyl-4-(2-methylproprionyloxy)-cinnamic acid (**9**), kaempferide (**10**), 3-hydroxy-2,2-dimethyl-8-prenyl-2H-1-benzopiran-6-propenoic acid (**11**), 3,5-diprenyl-4-hydroxycinnamic acid, Artepillin-C (**12**) and 2,2-dimethyl-8-prenyl-2H-1-benzopyran-6-propenoic acid (**13**). AU corresponds to Absorbance Units.

It was possible to identify the cinnamic aldehyde at 9.5 min in *C. verum* hydroethanolic extract (Figure 1). Moreover, it was possible to identify some phenolic acids and derivatives and flavonoids, such as kaempferol and kaempferide BGP hydroethanolic extract (Figure 2).

### 2.3. Minimum Inhibitory (MIC) and Minimum Bactericidal (MBC) Values

It was not possible to identify MIC values because of the intense color of the tested extracts. However, the MBC of *C. verum* hydroethanolic extract was 2.2–4.4 mg/mL against *A. baumannii* and 0.5–4.4 mg/mL against *P. aeruginosa*. Furthermore, MBC values of BGP hydroethanolic extract was 5–10 mg/mL against *A. baumannii*, and 1.2–20 mg/mL against *P. aeruginosa*.

### 2.4. Combined Extracts’ Synergistic Effect

The combination of *C. verum* and BGP hydroethanolic extracts showed additive values for all tested strains of *A. baumannii* and *P. aeruginosa*, except of two concentrations on A2 and P1 clinical strains as calculated with the fractional bactericidal concentration (FBC) index, (Table 1).

### 2.5. Antibiofilm Effect (Preventing Biofilms Formation)

**Figure 3:**
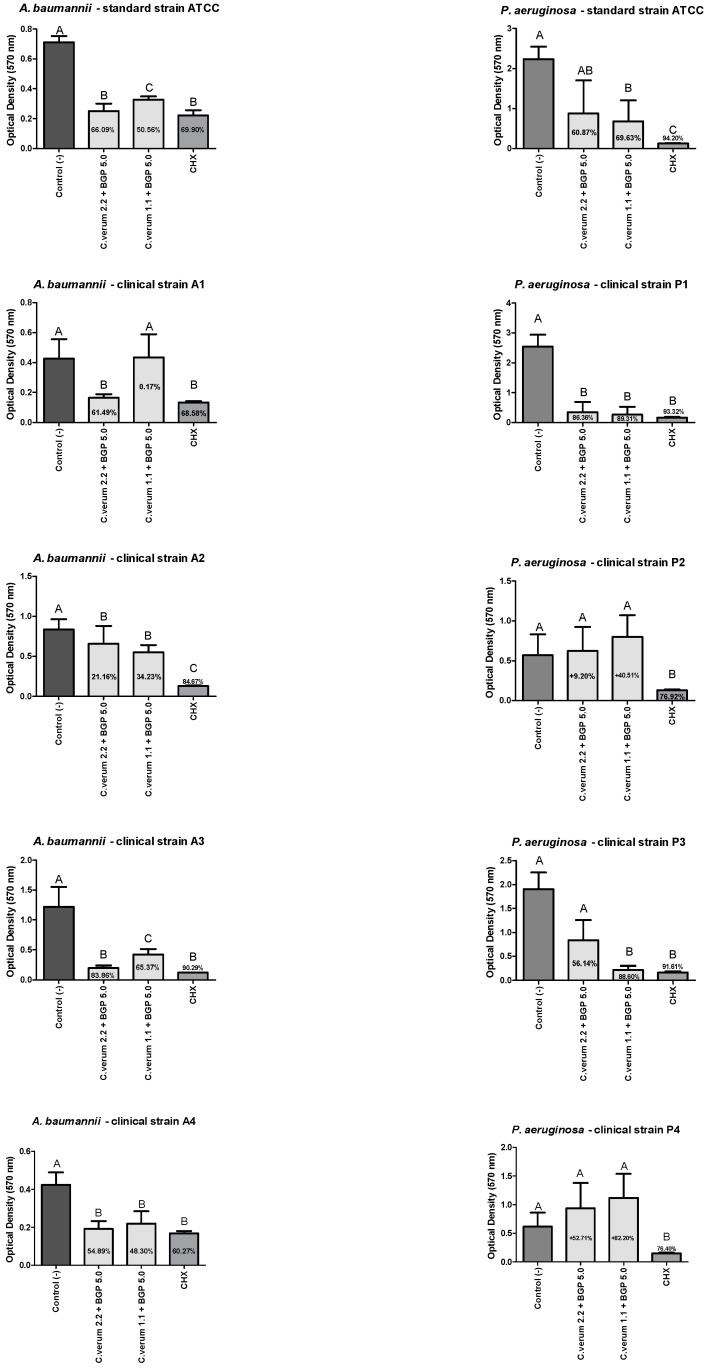
*A. baumannii* and *P. aeruginosa* biofilm load reduction after treatment with the combined extracts of *C. verum* and *BGP* to prevent biofilm formation. Legend: Different uppercase letters indicate a statistically significant difference.

The combined extract of 2.2 mg/mL *C. verum* + 5.0 mg/mL BGP was as effective as the CHX group in preventing a biofilm formation of all *A. baumannii* strains except of A2 strain without a statistically significant difference. However, CHX was more effective than the combined extract of 2.2 mg/mL *C. verum* + 5.0 mg/mL BGP against A2 strain. Still, the combined extract of 1.1 mg/mL *C. verum* + 5.0 mg/mL BGP was effective in preventing a biofilm formation of all *A. baumannii* strains except of A1 strain, with a statistically significant difference of the negative control group (Figure 3). 

The combined extract of 2.2 mg/mL *C. verum* + 5.0 mg/mL BGP was as effective as the CHX group in preventing a biofilm formation of *P. aeruginosa* clinical strain P1 without a statistically significant difference and as effective as the combined extract of 1.1 mg/mL *C. verum* + 5.0 mg/mL BGP without a statistically significant difference of the negative control group against *P. aeruginosa* ATCC strain. However, the combined extract of 1.1 mg/mL *C. verum* + 5.0 mg/mL BGP was effective in preventing a biofilm formation of all *P. aeruginosa* strains except of P2 and P4 strains with a statistically significant difference of the negative control group (Figure 3).

### 2.6. Antibiofilm Effect (against Mature Biofilms)

**Figure 4:**
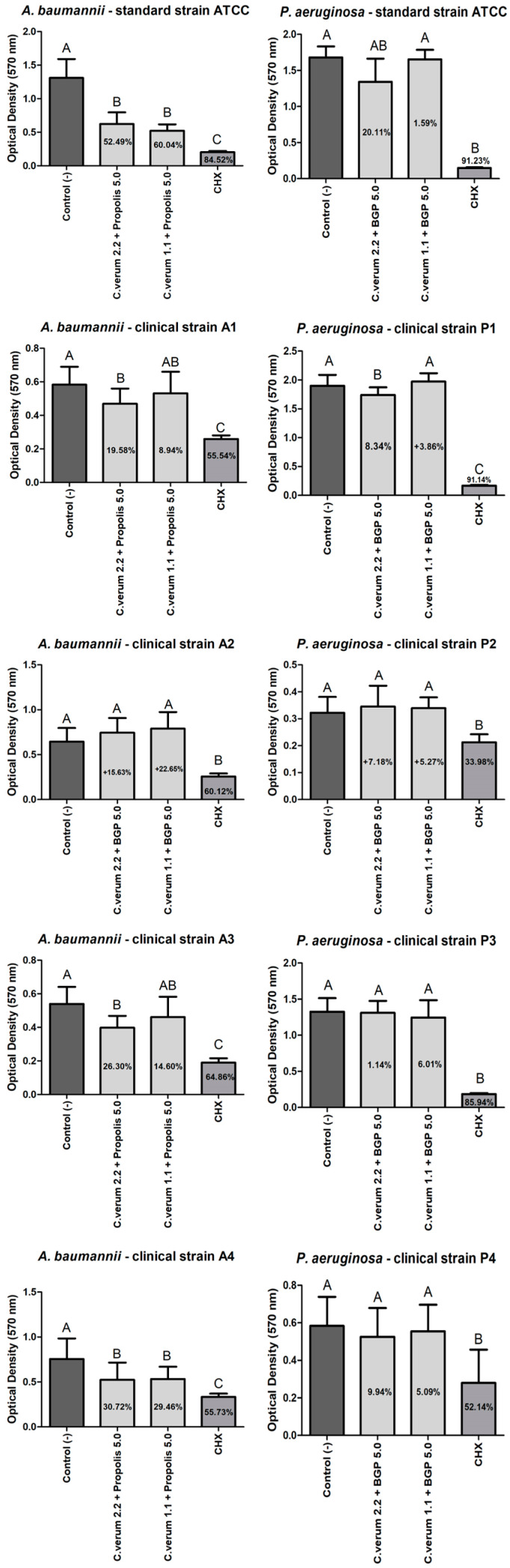
*A. baumannii* and *P. aeruginosa* biofilm load reduction after treatment with the combined extracts of *C. verum* and BGP for 5 min to reduce mature biofilm formation. Legend: Different uppercase letters indicate a statistically significant difference.

After 5 min, the combined extract of 2.2 mg/mL *C. verum* + 5.0 mg/mL BGP was effective in the mature biofilm reduction of all *A. baumannii* strains except for the A2 strain with a statistically significant difference of the negative control group; however, the combined extract of 1.1 mg/mL *C. verum* + 5.0 mg/mL BGP was effective in the mature biofilm reduction of *A. baumannii* ATCC and A4 clinical strains with a statistically significant difference of the negative control group. Still, the combined extract of 2.2 mg/mL *C. verum* + 5.0 mg/mL BGP was effective in the mature biofilm reduction of *P. aeruginosa* clinical strain P1 with a statistically significant difference of the negative control group. Conversely, the combined extract of 1.1 mg/mL *C. verum* + 5.0 mg/mL BGP was not effective in mature *P. aeruginosa* biofilm reduction (Figure 4).

**Figure 5:**
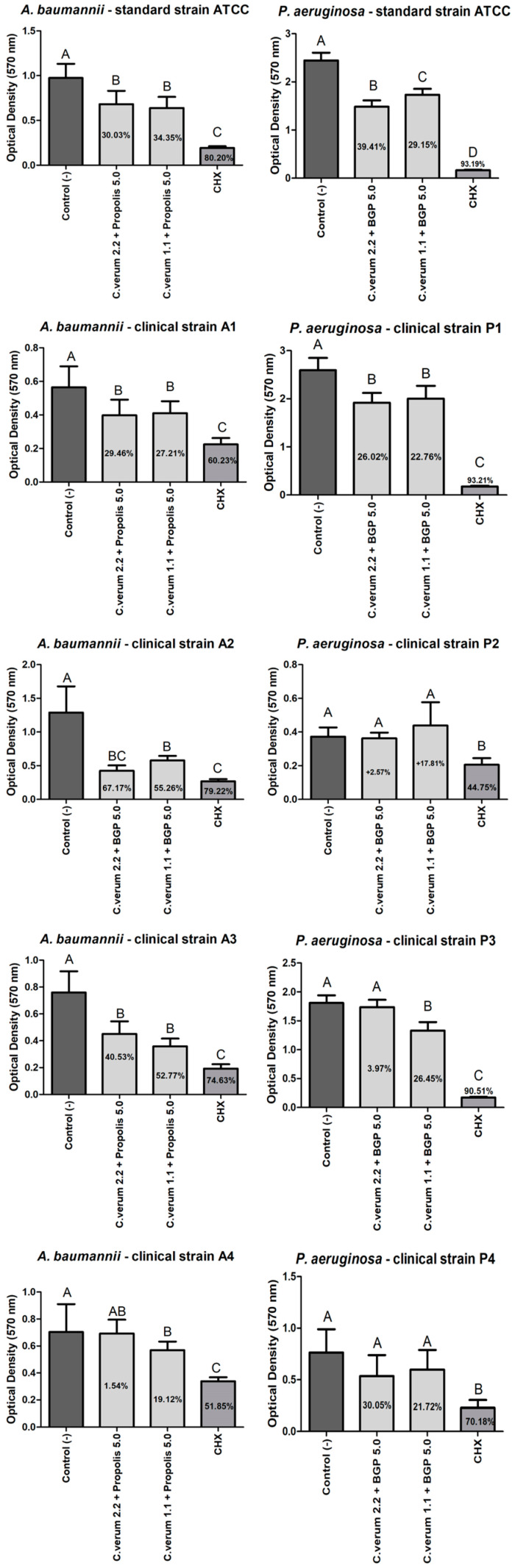
*A. baumannii* and *P. aeruginosa* biofilm load reduction after treatment with the combined extracts of *C. verum* and BGP for 30 min to reduce mature biofilm formation. Legend: Different uppercase letters indicate a statistically significant difference.

After 30 min, the combined extract of 2.2 mg/mL *C. verum* + 5.0 mg/mL BGP was effective in the mature biofilm reduction of all *A. baumannii* strains except of the A4 clinical strain with a statistically significant difference of the negative control group, and the combined extract of 1.1 mg/mL *C. verum* + 5.0 mg/mL BGP was effective in the mature biofilm reduction of all *A. baumannii* tested strains with a statistically significant difference of the negative control group. Furthermore, the combined extract of 2.2 mg/mL *C. verum* + 5.0 mg/mL BGP was effective in the mature biofilm reduction of *P. aeruginosa clinical* ATCC and the P1 clinical strains, and the combined extract of 1.1 mg/mL *C. verum* + 5.0 mg/mL BGP was effective in the mature biofilm reduction of *P. aeruginosa clinical* ATCC and the P1 and P3 clinical strains (Figure 5).

### 2.7. Cytotoxicity of Plant Extracts on Human Keratinocytes (HaCaT)

**Figure 6:**
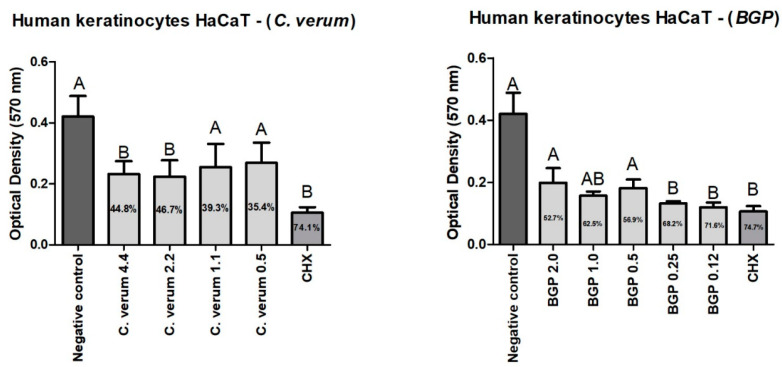
Human keratinocytes (HaCaT) viability reduction after 24 h of contact with *C. verum* and BGP hydroethanolic extracts. Values expressed in mg/mL; different uppercase letters indicate a statistically significant difference.

All *C. verum*-hydroethanolic-extract-tested concentrations were biocompatible; however, at 1.1 and 0.5 mg/mL they were more biocompatible than 0.12% CHX without a statistically significant difference of the control group (DMEM). In addition, all BGP-hydroethanolic-extract-tested concentrations were biocompatible; however, at 20, 10 and 5 mg/mL they were without a statistically significant difference of the control group (DMEM) (Figure 6).

## 3. Discussion

The synergy of different plant extracts was tested before and was effective against different microorganisms [22,23]. The main objective of this study was to evaluate the synergistic antibiofilm action of the produced and phytochemically characterized extracts of *C. verum* and BGP propolis against multidrug-resistant clinical strains of *A. baumannii* and *P. aeruginosa*. The combined effect of the tested extracts was effective in preventing biofilm formation and in reducing mature biofilms; thus, the null hypothesis was rejected. 

In the present study, cinnamic aldehyde was identified among the main components of the *C. verum* extract at 9.5 min. This identification agrees with the findings of another study that found the same component in the same extract at 4.06 min [24]. In addition, the phytochemicals in the BGP extract identified in the present study were caffeic, p-coumaric and other cinnamic acids and derivatives as well as flavonoids such as kaempferol and kaempferide. Such findings are in line with another study in the literature, in which p-coumaric acid, kaempferol and cinnamic acid were found in propolis extract [25]. The rich composition of propolis in secondary metabolites promotes this bee product as a promising substance to act against multidrug-resistant and anaerobic bacteria, since the action of the compounds together can hinder the emergence of microbial resistance [20,26].

In the present study, 100% of the *P. aeruginosa* isolates were resistant to meropenem and 75% to imipenem, and, furthermore, 100% of the clinical isolates of *A. baumannii* were resistant to carbapenems as found in Table 1. In the study of [27], during the collection of bacterial isolates from patients with pneumonia, 55.6% and 77.8% of the clinical strains of *P. aeruginosa* were resistant to meropenem and imipenem, respectively, and 100% of the clinical strains isolated from *A. baumannii* were resistant to meropenem and imipenem. 

In the present study, the *C. verum* hydroethanolic extract’s MBC value ranged from 2.2 to 4.4 mg/mL for A. *baumannii* and from 0.5 to 4.4 mg/mL for *P. aeruginosa*. In the literature, the MIC and MBC values of the cinnamon essential oil were 0.25 and 0.50 mg/mL, respectively, and they presented potent activity against *Staphylococcus aureus*, *Escherichia coli*, *P. aeruginosa* and *A. baumannii* with an inhibition zone reaching 16 mm against *P. aeruginosa* and 27.8 mm against multidrug-resistant clinical strains of *A. baumannii* [28]. Moreover, the BGP hydroethanolic extract MBC value was 17.01% on strains of *P. aeruginosa* and *A. baumannii*. The present study is in line with the results found in the literature, as the MBC value of propolis ranged from 0.12% to 2.01% on *P. aeruginosa* and *A. baumannii*. 

The application of isolated substances, whether of plant or synthetic origin, raises the hypothesis of the emergence of microbial resistance over the years, and the study of the combination of substances has gained strength in the literature. To the best of our knowledge, there is no study found in the consulted literature that evaluated the combination of *C. verum* and BGP hydroethanolic extracts against planktonic cultures of *A. baumannii* and *P. aeruginosa*. In the present study, sixteen additive combinations (FBC Index > 0.5 and <1) and two synergistic (FBC Index < 0.5) combinations were found against *A. baumannii* and *P. aeruginosa* with significant reductions in the MBC values of up to forty times. 

Regarding the results of the antibiofilm action of the extracts, it was possible to observe that the combined extracts promoted higher percentages in preventing biofilms’ formations than reducing the mature biofilms; such findings glimpse the prophylactic potential of these plant products combined in antiseptic formulations. The combined extract of 2.2 mg/mL *C. verum* + 5.0 mg/mL BGP inhibited the biofilm formation of the clinical strain of *A. baumannii* A3 by up to 83.86% with a performance comparable to chlorhexidine digluconate, which presented a percentage of 90.29%. Furthermore, the same extract promoted a reduction of 67.17% of the clinical strain of *A. baumannii* A2, also with a similar action to chlorhexidine gluconate (79.22%). This may be explained by the cinnamon essential oil that acts to inhibit and eradicate the biofilm of clinical strains of *A. baumannii* with reductions of up to 71% of the biofilm formed at a concentration of 1 mg/mL [29].

Against *P. aeruginosa*, the biofilm formation of the clinical strain P1 was reduced by the effect of the combined extract of 2.2 mg/mL *C. verum* + 5.0 mg/mL BGP by up to 86.36%; the combined extract of 1.1 mg/mL *C. verum* + 5.0 mg/mL BGP by up to 89.31%; and P3 was reduced by the effect of the combined extract of 1.1 mg/mL *C. verum* + 5.0 mg/mL BGP by up to 88.60%. In the study of Kalia et al. (2015) [30], the essential oil of *C. verum* promoted an inhibition of *P. aeruginosa* biofilm with a reduction of up to 54% in the production of polymeric substrate when applied at a concentration of 0.2 μL/mL. The capacity of *Cinnamomum* spp. to act against *P. aeruginosa* biofilm is attributed to its mechanism that ranges from the disruption of the cytoplasmic membrane, an ability to interfere with the quorum sensing system to inhibit biofilm formation at sub-inhibitory concentrations [31,32]. In the literature, the isolated propolis ethanol extract was able to promote a decrease in the release of eDNA from *P. aeruginosa* and, consequently, affect the stability of the biofilm [33].

Conversely, evaluating the results on clinical strains P2 and P4, no combination promoted biofilm reductions. For further studies, phenotypic and genotypic analyzes are suggested for a better characterization of the P2 and P4 biofilm. Other combinations of alternative therapies can also be considered for future analysis, such as the combination of extracts with antibiotics. Moreover, it was not possible to compare these results with others in the literature, as no studies were found in the consulted literature on the antibiofilm action of the combination of *C. verum* and Brazilian green propolis hydroethanolic extracts.

The cytotoxicity analyzes were carried out with the aim of verifying the potential interference of plant extracts in the viability of human cells. When evaluating the cytotoxicity of plant extracts, it was noted that chlorhexidine gluconate 0.12% (a gold standard in oral antisepsis) has greater cytotoxicity on human keratinocytes than any concentration of the extracts that were analyzed. In the evaluation of the *C. verum* extract, the percentages of cell viability in all analyzed concentrations were above 50%. Only higher concentrations (0.44 and 0.22%) differed from the cell growth control. The results of the present study are in line with the findings in the literature that found that no cytotoxic effect of *C. verum* essential oil (0–1000 mg/mL) was detected on human keratinocytes [34]. The vegetable product was kept in contact with the cells for 24 h under the same experimental conditions. When evaluating the cytotoxicity of the green propolis extract, it was observed that the lowest concentrations produced greater toxicity than the highest concentrations (2.01%, 1.0% and 0.5%). However, again, it is possible to observe that the chlorhexidine gluconate shows the highest percentage of reduction in keratinocyte viability (74.7%) which demonstrates a cytotoxic potential in human epithelial cells. In the study by Bae et al. (2022), the authors also verified a slight decrease in cell viability in HaCaT strains when Korean propolis ethanolic extract was applied [35].

Finally, faced with the challenge of the multidrug resistance that the studied bacteria currently present, the findings are of great contribution to science, since the synergism of compounds can occur through several mechanisms bypassing microbial resistance [36]. This study suggests the use of combined extracts of *C. verum* and Brazilian green propolis against multidrug resistance strains of *A. baumannii* and *P. aeruginosa* that are present in the oral cavity of hospitalized patients, considering these extracts as potential antiseptic agents for future dental formulations that can be aimed at prophylaxis and used for control of nosocomial infections caused by these microorganisms. 

## 4. Materials and Methods

### 4.1. Plants and Propolis Extracts

The *C. verum* extract was prepared from cinnamon bark or cinnamon removed from the trunk purchased commercially (Com-sciência Saúde, São José dos Campos, SP, Brazil) from Saigon (Vietnam) and the Brazilian green propolis (BGP) of *Baccharis dracunculifolia* was prepared from raw propolis provided by the supplier Apis Brasil (Pindamonhangaba, SP, Brazil). The vehicle chosen for extraction was absolute ethanol (ethyl alcohol 99.5%—Merck Darmstadt, Germany) and ultrapure water obtained in the Milli-Q^®^ system (EtOH: H_2_O/50:50), following the proportion 30 g of raw material for each 100 mL of the vehicle, with an extraction time of 48 h. Finally, the extracts were filtered in two stages: filtration to remove solid residues (paper filter with microholes) and filtration for sterilization (0.22 µm membrane filter). The raw propolis has the following characteristics (provided by quality control laboratory from Apis Brasil): total phenolic compounds: 8.50% (*w*/*w*), flavonoids 3.00% (*w*/*w*), wax 13.80% (*w*/*w*), ash 3.90% (*w*/*w*), insoluble residues in ethanol 36.05% (*w*/*w*) and soluble solids in ethanol 43.01% (*w*/*w*) in accordance with the Brazilian norms of the Ministry of Agriculture [37].

### 4.2. Content of Soluble Solids

Six empty beakers of 25 mL were weighed, and the weights recorded. Then, 5 mL of the extracts were pipetted into each one of them (triplicate of each extract), and then they were dried at 80 °C between 24 and 48 h. After drying, the beakers were placed in a desiccator until completely cooled and then weighed. Finally, the content of soluble solids presents in plant and propolis extracts was quantified using mathematical formulas based on the values of the weight of the beakers.

### 4.3. High-Performance Liquid Chromatography Analysis of the Plant Extracts

High-performance liquid chromatography (HPLC) was used to characterize and quantify the content of markers in the natural extracts. The analysis was performed in a high-efficiency liquid chromatograph; the stationary phase was a LiChrospher^®^ RP-18 HPLC column, 5 μm particle size, L × I.D. 12.5 cm × 4.6 mm from Merck, Darmstadt, Germany. We used a photodiode detector (HPLC-DAD) and an automatic injector model D-7000 Merck-Hitachi (Merck KGaA, Darmstadt, Germany). The chromatographic conditions were mobile phase: water–formic acid solution (PA, Merck, Darmstadt, Germany) diluted in the ratio of 95:5 (solvent A) and methanol HPLC grade Merck (Darmstadt, Germany, solvent B). The flow was 1 mL/min and a linear gradient starting with 0% B, ending with 70% B, in a 50 min run time. The detection wavelengths were 280 and 340 nm [20,22]. Quantification of the polyphenols in BGP extract was performed using standards with the same UV spectrum. P-coumaric acid, prenylated p-coumaric acids, such as 3,5-diprenyl-4-hydroxycinnamic acid (12), and cinnamic acid derivatives were quantified using individual calibration curves to p-coumaric acid (3) (Sigma-Aldrich Chemistry, St. Louis, MO, USA), while caffeic and caffeoylquinic acids were quantified using caffeic acid (2, Sigma-Aldrich). Standards for the quantification of kaempferol (5), kaempferide (10) and 2,2-dimethyl-2H-1-benzopyran-6-propenoic acid (6) were not available [38,39]. 

### 4.4. Inoculum Preparation

Four multidrug-resistant clinical strains of *A. baumannii* (A1, A2, A3 and A4) and four multidrug-resistant clinical strains of *P. aeruginosa* (P1, P2, P3 and P4) were used in this study (Table 2); the strains were obtained from the clinical laboratories of the Policlin (Pliclin Saúde group, São José dos Campos, SP, Brazil) and ValeClin Clinical Analysis Laboratory (ValeClin Clinical Analysis Laboratory, São José dos Campos, SP, Brazil). The resistance profile of each strain was obtained through the automated MicroSCAN AutoSCAN 4^®^ system. Additionally, the reference strains of *A. baumannii* (ATCC 19606) and *P. aeruginosa* (ATCC 15442) were used in this study. The bacterial strains were cultivated (37 °C/24 h) in BHI agar (Brain Heart Infusion—Kasvi, São José dos Pinhais, PR, Brazil) for subsequent preparation of microbial suspensions in sterile saline solution (0.9% NaCl).

### 4.5. Minimum Inhibitory (MIC) and Minimum Bactericidal (MBC) Concentrations 

Microdilution method was used according to Clinical and Laboratory Standards Institute (CLSI) documents M7-A9 to determine the minimum inhibitory (MIC) and minimum bactericidal (MBC) concentrations of the extracts for each bacterial strain. Microbial suspensions were prepared in sterile saline solution (0.9% NaCl) with turbidity adjusted to 10^8^ colony-forming units per milliliter (CFU ml^−1^) in a spectrophotometer (Micronal, São Paulo, SP, Brazil). The parameters used were λ = 625 nm and optical density (OD) of 0.08 ±  0.02 for both bacteria tested. From these stock solutions, dilutions of 10^6^ CFU ml^−1^ were prepared for testing in planktonic cultures. In a different 96-well microplates (TPP, Trasadingen, Switzerland) for each bacterial strain, 100 µL/well of Mueller Hinton Broth (Himedia) was added. Next, 200 µL of one of the extracts was added in the first well, where 10 serial dilutions were carried out. Then, 100 µL/well of the respective bacterial inoculum was added. The microplates were incubated for 24 h at 37 °C. The MIC of each extract was determined, by eye, in the first well without turbidity, following the well with apparent microbial growth. In addition, analyses of the vehicle (EtOH:H_2_O/50:50) were performed to verify whether it interferes with the antimicrobial action of plant extracts. To determine the MBC, the contents of the wells were seeded onto BHI agar. After incubation (37 °C/24 h), the MBC was determined as the lowest concentration of the extract that inhibited total microorganism growth.

### 4.6. Combined Extracts Synergistic Effect

The checkerboard technique was used according to Moreno et al. [40] with modifications; this method is based on the Microdilution method of the Clinical and Laboratory Standards Institute. A new bacterial inoculum was prepared and standardized in saline solution (1 × 10^6^ cells/mL). Eight concentrations of each extract were prepared based on MIC value of each extract for each bacterial strain being diluted in Mueller Hinton broth [41]. Then, 50 µL of the first extract (*C. verum* hydroethanolic extract) was added along the *x*-axis (horizontal) of the microplate, and 50 µL of the second extract (BGP hydroethanolic extract) was added to the *y*-axis (vertical) of the microplate. Saline solution and culture medium were used as control groups. Lastly, 100 µL of the standardized inoculum of each strain was added totaling 200 µL/well. The microplates were incubated at 37 °C/24 h for further visual reading. To evaluate the synergistic effect of the extracts, the fractional inhibitory concentration (FIC) index was adopted, which classifies the combinations as synergistic, additive, indifferent or antagonistic. The FIC index was calculated using the formula:

FIC index = FIC 1st + FIC 2nd = (MIC of the 1st extract in combination / MIC of the 1st extract alone) + (MIC of the 2nd extract in combination / MIC of the 2nd extract alone).

The combination was considered synergistic when FIC ≤ 0.5, additive when FIC > 0.5 and ≤1.0, indifferent when FIC > 1 and ≤ 4 and antagonist when FIC > 4.0. The same formula was used to calculate the fractional bactericidal concentration index (FBC), using MBC values instead of MIC.

### 4.7. Antibiofilm Action

Bacterial inoculum of each bacterial strain was prepared and standardized in a spectrophotometer (10^7^ cells/mL); then, it was added to microplates (100 μL/well) already containing culture medium, totaling 200 μL/well. Then, the plates were treated with two different methods.

In the first method, to evaluate the capacity of the extract on preventing biofilms formation [32], the extracts (n = 10) were added together with the standardized inoculum (1 × 10^7^ cells/mL) and incubated in 96-well plates at 37 °C for 48 h, changing the broth (diluted and combined plant extracts in the chosen concentrations) after 24 h.

In the second method, to evaluate the actions of the extract against mature biofilms, the plates were incubated at 37°/48 h for biofilm formation [12], with replacement of the broth after 24 h of incubation. Then, they were exposed for 5 min or 30 min to the diluted and combined plant extracts in the chosen concentrations.

The combinations selected to proceed with the biofilm analysis were named according to the wells identified in the Checkerboard experiment: combination 2.2 mg/mL *C. verum* + 5.0 mg/mL BGP and combination 1.1 mg/mL *C. verum* + 5.0 mg/mL BGP. 

For both methods, after the incubation time, the broth was removed, and the wells were washed-out with saline solution to eliminate non-adherent cells that suffered from the action of the extracts. The cell viability test of bacterial cells was performed in which 100 μL of MTT solution (3-(4,5-dimethylthiazol-2-yl)-2,5-diphenyltetrazolium bromide) (Sigma-Aldrich, Jurubatuba, Brazil) were added in each well and the plates were incubated in the dark at 37 °C for 1 h. After the incubation period, the MTT solution was removed, followed by the addition of 100 μL of dimethylsuffoxide (DMSO). The plates were again incubated at 37 °C for 10 min and placed in the Shaker under constant agitation for 10 min. Then, the optical densities (OD) were obtained using a microplate reader at 570 nm, and the OD obtained were converted into percentage of metabolic activity [42]. Culture medium was used as a negative control group and 0.12% chlorhexidine gluconate as a positive control group for all biofilm analyses.

### 4.8. Cytotoxicity of Plant Extracts on Human Keratinocytes (HaCaT)

The analysis of the cytotoxicity of the extracts was carried out on human keratinocytes (HaCaT) strains from the Rio de Janeiro Cell Bank—Associação Técnico Científica Paul Ehrlich (APABCAM—RJ). The cell lines were cultured in Dulbecco’s modified Eagle’s medium (DMEM—LGC Biotecnologia, Cotia, Brazil), with a high concentration of glucose, supplemented with 10% fetal bovine serum (FBS) (Invitrogen, New York, NY, USA) and maintained in cell culture flasks (TPP, Switzerland), incubated at 37 °C, atmospheric humidity and 5% CO_2_ for 24 h. When they reached the required amount, the cells were sent to the respective tests. For this purpose, the cell monolayer was disaggregated from the floor of the culture flask with trypsin.

The cytotoxicity of the extracts was verified using the MTT colorimetric test, evaluating the cellular enzymatic activity. Cultures were transferred to 96-well microplates (TPP) at a concentration of 2 × 10^4^ viable cells and cultivated in 200 µL of DMEM medium + 10% FBS. The plates were incubated (37 °C, 5% CO_2_) for 24 h for cell adherence. Then, the cells were exposed for 24 h to the extracts and controls according to the groups: *C. verum* hydroethanolic extract (of 4.4, 2.2, 1.1 and 0.5 mg/mL), BGP hydroethanolic extract (of 20, 10, 5, 2.5 and 1.25 mg/mL), DMEM as a negative control group, and 0.12% chlorhexidine gluconate (CHX) as a positive control group. Concentrations of plant extracts were based on the MBC results found in microbiological experiments. Then, the cells were submitted to the MTT test, using a suspension of 0.5 mg of MTT powder (Sigma-Aldrich, Jurubatuba, Brazil) in 1 mL of DMEM + 10% FCS. The solution was transferred to 96-well microplates in a volume of 100 µL/well, followed by incubation in the dark (37 °C, 5% CO_2_) for 4 h. Subsequently, the solution was discarded and 100 µL/well of dimethylsulfoxide (DMSO–Sigma) were added. After incubation for 10 min and agitation in a shaker, the absorbance of the wells was read in a microplate reader with a wavelength of 570 nm. The optical densities (OD) obtained were converted into percentage of cell viability.

### 4.9. Statistical Analysis

Data were analyzed with normality tests: Shapiro–Wilk, Kolmogorov–Smirnov and D’Agostino and Pearson omnibus. Normal data were analyzed with ANOVA and Tukey’s test, otherwise, with the Kruskal–Wallis test and Dunn’s test. The GraphPad Prism 5.0 program was used, considering a significance level of 5%. 

## 5. Conclusions

- *C. verum* and BGP hydroethanolic extracts have bactericidal and antibiofilm action against multidrug resistant strains of *A. baumannii* and *P. aeruginosa*.
- The combined extracts were capable of expressively inhibiting the formation of *A. baumannii* and *P. aeruginosa* biofilms (prophylactic effect) acting similarly to 0.12% chlorhexidine gluconate.

## Figures and Tables

**Table 1:** Minimum bactericidal concentration (MBC) values in mg/mL, in addition to the combined effect of *C. verum* and BGP hydroethanolic extracts against *A. baumannii* and *P. aeruginosa* strains.

Bacterial Strains	Isolated Extract MBC Value (mg/mL)		Combined Concentrations (mg/mL)		FBC Index	Reduction in MBC		Effect
	*C. verum*	BGP	*C. verum*	BGP		*C. verum*	BGP	
*A. baumannii* ATCC	2.2	10	1.1	5	1.00	2×	2×	Add
			0.5	5	0.72	4×	2×	Add
A1	4.4	5	-	-	-	-	-	-
A2	4.4	10	2.2	5	1.00	2×	2×	Add
			1.1	5	0.75	4×	2×	Add
			0.5	5	0.61	8×	2×	Add
			0.2	5	0.54	20×	2×	Add
			0.1	5	0.53	40×	2×	Add
			2.2	2.5	0.75	2×	4×	Add
			1.1	2.5	0.50	4×	4×	Syn
			2.2	1.2	0.62	2×	8×	Add
A3	4.4	10	2.2	5	1.00	2×	2×	Add
			1.1	5	0.75	4×	2×	Add
A4	4.4	5	-	-	-	-	-	-
*P. aeruginosa* ATCC	0.5	1.2	0.2	0.6	0.90	2×	2×	Add
P1	1.1	2.5	0.5	1.2	0.93	2×	2×	Add
			0.2	1.2	0.66	5×	2×	Add
			0.5	0.07	0.47	2×	34×	Syn
P2	4.4	20	2.2	10	1.00	2×	2×	Add
P3	0.5	1.2	-	-	-	-	-	-
P4	4.4	20	2.2	10	1.00	2×	2×	Add

Legend: Add: Additive effect. Syn: synergistic effect.

**Table 2:** Antibiotic resistance profile of clinical strains of *A. baumannii* and *P. aeruginosa* provided by the Policlin Group Laboratory and Valeclin Clinical Analysis Laboratory.

Antibiotic	*A. baumannii* Clinical Strains			
	A1	A2	A3	A4
Ertapenem	Res	Res	Res	Res
Imipenem	Res	Res	Res	Res
Meropenem	Res	Res	Res	Res
	*P. aeruginosa* clinical strains			
	P1	P2	P3	P4
Amikacin	Res	Res	Sen	Res
Aztreonam	Res	Inter	Res	Res
Cefepime	Res	Res	Res	Sen
Ceftazidime	Res	Res	Res	Sen
Ciprofloxacin	Res	Sen	Res	Res
Colistin	Inter	Res	-	-
Gentamicin	Res	Res	Sen	Res
Imipenem	Res	Res	Inter	Res
Levofloxacin	Res	Sen	Res	Res
Meropenem	Res	Res	Res	Res
Piperacillin/Tazobactam	Res	Inter	Inter	Sen
Polymyxin B	-	-	-	Sen
Tobramycin	Res	-	-	Res

Legend: Resistant = Res; Intermediary = Inter; Sensitive = Sen.

## Data Availability

The data used to support the findings of this study are available upon request with the corresponding author d.d.s.amjad@gmail.com.

## Sample Availability

The samples of this study are available upon request with the corresponding author d.d.s.amjad@gmail.com.
